# Chronotype Profile, Stress, Depression Level, and Temporomandibular Symptoms in Students with Type D Personality

**DOI:** 10.3390/jcm11071886

**Published:** 2022-03-28

**Authors:** Magdalena Gębska, Bartosz Dalewski, Łukasz Pałka, Łukasz Kołodziej, Ewa Sobolewska

**Affiliations:** 1 Department of Rehabilitation Musculoskeletal System, Pomeranian Medical University, 70-204 Szczecin, Poland; mgebska@pum.edu.pl (M.G.); lukas@hot.pl (Ł.K.); 2 Department of Dental Prosthetics, Pomeranian Medical University, 70-204 Szczecin, Poland; bartosz.dalewski@pum.edu.pl (B.D.); rpsobolewski@wp.pl (E.S.); 3 Private Dental Practice, 68-200 Zary, Poland

**Keywords:** chronotype, circadian rhythm, personality, temporomandibular disorders, stress, depression

## Abstract

Background: Despite a growing interest in the types of human circadian activity, different chronotypes and personality-related issues have been rarely studied. It has already been emphasized that ‘stress personality’ is considered a risk factor for certain psychosomatic diseases and may be a temporomandibular disorders (TMDs) predictor. Therefore, an attempt has been made to analyze the chronotypes, stress levels, stress factors, and the occurrence of depression and TMDs in students with type D personalities. People with this personality trait tend to experience negative emotions more—depression, anxiety, anger, or hostility—yet may have a negative image of themselves and report somatic complaints. Aim: The aim of this study was to analyze the importance of the chronotype profile for the level of stress perceived, as well as for the occurrence of depression and TMDs in people with type D personalities. Material and Methods: The study has been conducted on a group of 220 physical therapy students. The study group G1 consisted of 110 participants with type D personalities, the control group G2 consisted of the same number of participants without the stress personality. All participants have been analyzed for the chronotype (MEQ), stress perception (PSS10), the occurrence of depression (Beck scale-BDI), the occurrence of TMDs symptoms and have completed the stress factor assessment questionnaire during the study, followed by DS14 questionnaire—a tool for assessing the prevalence of type D personality. Results: In students with type D personalities (G1), the definitely evening and evening chronotypes have been significantly more predominant than in the control group (G2). A significantly higher number of stressors and TMDs symptoms have been observed in the respondents from the G1 group than in the control group (<0.001). Univariate logistic regression analysis showed that type D personality was strongly associated with a more frequent occurrence of all TMD symptoms. Additionally, a significant influence of the evening chronotype on the occurrence of type D personality was observed. Among the potential confounding variables, female gender and a mild and moderate degree of depression have an impact on the occurrence of type D personality (*p* < 0.05). In the multivariate model, adjusted with the above-mentioned factors, an increased risk of the type D personality trait was found. Conclusion: The evening chronotype and type D personality may imply greater feelings of stress, greater depression, and more frequent symptoms of TMDs in young adults.

## 1. Introduction

In the human body, a number of processes take place in a cyclical manner. The most common and best-known biological rhythms are the daily (circadian) rhythms, which run in cycles that last for about 24 h, with a typical example of the rhythm of sleep and wake [1]. The central circadian clock (oscillator) is considered to control behavioral and humoral rhythms in living organisms including humans. It is located in the suprachiasmatic nucleus of the hypothalamus, which receives the stimuli of light and darkness directly from the retina and correlates them with the preferences concerning sleep time [2]. Circadian oscillators are located in the cells of the central nervous system and in most other cells in the body [3,4]. The molecular foundation of the biological clock is a periodical expression of a wide array of the so-called clock genes that control the regularity of life processes and the dependent production of clock proteins. The disturbances of the physiological circadian rhythms play a significant role in human pathology—they can be either the cause or the effect of a number of somatic and mental diseases [5].

The link between the circadian rhythm and certain diseases has led researchers to believe that rhythmic bodily activity is a symptom of good health and that circadian irregularities are harmful to health [6,7]. In fact, a number of studies have revealed that any irregularities in circadian rhythms can cause metabolic diseases such as diabetes, obesity [8], tumors [9], neurodegenerative diseases [10], and adverse cardiovascular consequences such as an increased risk of cardiovascular disease and stroke-induced mortality [11].

In humans, there are broad individual differences in circadian phases of sleep-wake rhythms, psychophysical activity, body temperature, cortisol, and melatonin secretion [12,13,14]. Specific preferences for falling asleep, waking up, and increased mental and physical activity have been the source for distinguishing various types of circadian activity, i.e., chronotypes [15]. These include: M (Morning, “larks”) and E (Evening, “owls”). The manifestation of a given chronotype depends on a number of parameters: genetic predispositions, age, sex, socioeconomic factors, exposure to light, the season of birth. The academic studies carried out so far have indicated a link between a given chronotype (M or E) and the sleep cycle and effectiveness, lifestyle regularity, level of anxiety, mood changes, and well-being [16,17]. The evening chronotype has been associated with an increased risk for mood disorders, such as major depressive disorder [18], bipolar disorder [19], and seasonal affective disorder [20]. Increasingly, evening chronotype has been linked with mental health problems beyond mood disorders such as attention deficits [21], anxiety [22], alcohol intake resulting in later dependence [23], and antisocial behaviors [24], suggesting that the evening chronotype may constitute a transdiagnostic risk factor more broadly [25].

Intricate connections between chronotype and personality traits have long been the subject of interest for researchers. Previous scientific reports have indicated that E types demonstrate certain traits and patterns in their behavior that are associated with adaptation difficulties and problems in coping with environmental and social requirements: higher levels of neuroticism, anxiety, and greater susceptibility to depression [26,27,28]. Moreover, certain authors point out the tendency for evening people to engage in bulimic behavior, binge eating, unfavorable health habits, and increased consumption of stimulants [29,30,31]. M types are believed to lead a “healthier” lifestyle than their evening counterparts, which results in better psychosomatic health. According to Tonetti et al. [32], the morning types scored significantly higher on the conscientiousness scale, while evening types on the scale of neuroticism. Greater emotional stability of the morning types has also been proven in the analysis carried out by Colin DeYoung et al. [33]. So far, four basic personality types: A (“coronary”), B (“neutral”), C (“cancer”), and D (“stress”) were described [25,26,32].

Yet, no studies have been performed linking chronotype and type D personality, which is the most recently distinguished type of personality, characterized by two dimensions: negative affectivity (NA) and social inhibition (SI). Individuals having this type of personality tend to experience negative emotions—depression, anxiety, anger, or hostility. They have a negative perception of themselves and report more complaints about somatic disorders. They reveal a tendency for social inhibition, i.e., avoiding contact with other people, avoiding potential threats resulting from social interactions. Type D personality shows certain similarities to the two personality dimensions that make up the “Big Five”, i.e., neuroticism and introversion. The above was proven in the research carried out by De Fruyt and Denollet. We can thus assume that the type D personality is an equivalent of neurotic introversion [34]. People with type D personalities are at increased risk of mental disorders such as anxiety, post-traumatic stress disorder, phobia, and depression [35,36,37]. Type D personality has been found to be a significant predictor of cardiovascular diseases, cancer, gastric ulcer, duodenal ulcer, and skin diseases [38,39,40,41]. As indicated by the studies conducted so far in the fields of dentistry and psychology, people with type D personalities are more likely to experience TMDs (temporomandibular disorders) symptoms, headaches, and shoulder girdle pain, which may indicate a predictive role of the stress personality in the development of said diseases [42,43,44]. Hence, we hypothesized that type D personality traits may be the contributing factor affecting chronotype profiles and the occurrence of psychosomatic disorders, including TMDs.

### Aim

The aim of the study was to analyze the types of circadian activity in two personality groups among the Polish population (with and without type D personality) and to study the link between the chronotype and the perception of stress and stressors, or the occurrence of depression and TMDs in people with a “stress” personality.

## 2. Materials and Methods

The study was carried out from October 2020 to October 2021 among 220 students studying physiotherapy at the Pomeranian Medical University in Szczecin. The study group (G1) consisted of 110 participants who were diagnosed with type D personality according to the DS-14 questionnaire. The control group (G2) involved the same number of participants without type D personality.

The inclusion criteria within the control group were as follows: a first-, second-, third- or fourth-year student of physical therapy studying within a hybrid system (stationary and remote); “no stress” personality diagnosed according to DS14, no history of neurological, mental or autoimmune diseases, age between 20 and 28 years; consent to participate in the study. Exclusion criterion: chronic diseases, including psychosomatic diseases, pregnancy. The presence of type D personality in the G1 group was the differentiating factor in the inclusion criteria in the G1 and G2 groups.

The study was approved by the Pomeranian University of Medical Science Institutional Ethical Committee (KB-0012/79/16).

### 2.1. Research Tools

(a) Psychological Questionnaire DS14 (Type-D scale). For assessing the presence of a stressful personality in this study the authors used the validated Polish adaptation of DS-14 [45].

It consists of 7 questions related to the tendency to experience negative emotions and 7 questions related to tendencies to refrain from expressing these emotions. Each statement was rated on a scale from 0 (false) to 4 (true). The theoretical range of scores for each dimension was 0 to 28 points. Classification of type D required obtaining at least 10 points in each of the two dimensions, i.e., NA and SI. The reliability coefficient of Cronbach′s alphas for the DS14 was 0.88.

(b) Morningness-Eveningness Questionnaire (MEQ) (Supplementary Materials). This scale is a widespread tool for assessing the chronotype. Polish validation data was not available.

It consists of 19 closed questions and was used to assess the chronotype. Each statement was rated on a scale of 1 to 5 points. By completing the questionnaire, the patient could get from 16 to 86 points. Depending on the total number of points obtained, you could classify one’s chronotype as definitely evening (16–30 points), moderately evening (31–41 points), intermediate (42–58 points), moderately morning (59–69 points), or definitely morning (70–86 points). The reliability coefficient of Cronbach′s alphas for the MEQ was 0.83 [46].

(c) Perceived Stress Scale (PSS-10) (Supplementary Materials). For assessing the stress level in this study the authors used the validated polish adaptation of PSS10 It contains 10 questions about different subjective feelings related to personal problems and events, behaviors, and ways of coping. The respondents provided their answers by entering the correct number (0—never, 1—almost never, 2—sometimes, 3—quite often, 4—very often). The overall score on the scale was the total of all points, the theoretical distribution of which was from 0 to 40. The higher the score, the greater the severity of the perceived stress. The general indicator after conversion to standardized units was interpreted according to the properties characterizing the sten scale (a scale of psychological test normalized so that the population mean is 5.5 and the standard deviation is 2. The scale has 10 units). Scores in the range 1–4 sten (0–13 points) were treated as low and in the range 7–10 sten (20–40 points) as high. Results between 5 and 6 sten (14–19 points) were considered average.

The reliability coefficient of Cronbach′s alphas for the PSS-10 was 0.76 [47].

(d) Beck Depression Inventory (BDI) (Supplementary Materials). Advantages of the inventory are its high internal consistency, high content validity, validity in differentiating between depressed and nondepressed subjects, and sensitivity to change. The original version has proven satisfactory psychometric properties, while the data on the Polish translation are still only preliminary, despite being frequently used in practice and research. The BDI consists of 21 questions. The participants could choose one of four answers to each question. Each answer was assigned a value of 0 to 3 points. The theoretical range of scores for each dimension was 0 to 63 points. Depending on the total number of points obtained, one could detect the absence of depression (0–11 points), mild depression (13–19 points), moderate depression (20–25 points), and severe depression (above 26 points). The reliability coefficient of Cronbach’s alphas for the BDI was 0.86 [48].

(e) Our own questionnaire related to TMDs symptoms and predisposing factors., i.e., headache, neck pain, shoulder girdle pain, TMJ (temporomandibular joint) pain, TMJ acoustic symptoms, increased masticatory muscle tension, TMJ locking, and teeth clenching and grinding (Supplementary Materials).

(f) Questionnaire for assessing 29 selected stressors during the course of studies.

### 2.2. Characteristics of the Studied Groups

A total of 148 women (67.2%) and 72 men (32.7%) participated in the study. The average age in the G1 group was 21.34 years (SD 2.13). This group consisted of 91 women (82.7%) and 19 men (17.2%). The G1 group consisted of 56%, 27%, 12%, and 25% of first-year, second-year, third-year, and fourth-year students, respectively. The G2 control group consisted of 110 students, the average age was 22.95 years (SD 6.11). The group consisted of 57 women (51.8%) and 53 (48.1%) men. The G2 group consisted of 40.9%, 38.2%, 12.7%, and 8.2% of first-year, second-year, third-year, and fourth-year students, respectively. There was no difference in the sex structure between the groups (*p* = 0.74). There was a difference in the sex distribution between the groups, females dominated in the group with type D personality (<0.001).

### 2.3. Statistical Analysis

Data are presented as the mean +/− SD for quantitative variables with a normal distribution and as the median. A quartile for ordinal and quantitative variables with the other-than-normal distribution. The qualitative variables were ordered and presented as the number of cases in a given group together with the percentage value. The Pearson Chi-square test was used to analyze the differences between the distribution of qualitative variables. In order to compare quantitative variables, we used the T-student test for normally distributed variables and the Mann-Whitney U test for variables with other-than-normal distribution [44]. In order to analyze the connection between type D personality considered as a dependent variable and the stress and circadian rhythm-related factors that subject to this analysis are considered independent variables, we constructed a logistic regression model which was presented as OR values with a 95% confidence interval (CI).

The reliability and validity of the applied tests were assessed using the Cronbach′s alpha calculation.

## 3. Results

Significantly higher chances for being diagnosed with definitely evening and moderately evening chronotypes have been observed among participants with personality type D (G1), as compared with the control group (G2). The frequencies of the occurrence of the morning chronotype did not differ significantly between the groups (Table 1).

A significant negative link has been observed between the chronotype order from evening to morning and the perceived stress scale PSS 10 (expressed both in sten and points), and the results indicated on the BDI depression scale (Table 2).

The most commonly reported stressors among participants were: sleep deprivation (63.6%), large amount of learning (59.1%), and tests and difficult exams (58.6%). On the contrary, the most frequently reported symptoms of TMDs and predisposing factors were: headache (60.5%), pain in the neck and shoulder girdle (50.5%), and teeth clenching (47.3%) (Table 3).

A significantly higher number of stressors and TMDs symptoms and predisposing factors have been observed among the respondents with D-type personality (G1) as compared with the control group (Table 4).

Univariate logistic regression analysis showed that type D personality was associated with a significantly higher incidence of all the analyzed TMDs. Furthermore, the following was more common among respondents with type D personality: sleep deprivation, disappointment with studies, critical comments from the lecturer, difficulties in establishing contacts, a sense of not being understood, “unexpected situations”, and “speaking during classes” (Table 5).

A significant influence of the 0 + 1 chronotype (definitely and moderately evening) on the occurrence of type D personality has been observed (Table 6). Among the potential confounding variables, a significant influence of the female gender and mild and moderate degree of depression on the occurrence of the D personality was observed. Whereas in the multivariate model, adjusted with the above-mentioned factors, an increased risk of type D personality has been observed in the case of the 3 + 4 chronotype (definitely and moderately morning), which turned out to be a significant factor for the occurrence of type D personality after being adjusted with the remaining variables.

## 4. Discussion

The chronotype is, above all, dependent on our biological clock and genetic predispositions [49,50]. Additional factors include personality and lifestyle [51,52]. According to the studies, a significantly higher chance of being diagnosed with definitely evening and moderately evening chronotypes has been observed among the students with type D personality, as compared with the control group. The frequencies of the occurrence of the morning chronotype have not differed significantly between the two groups. Conden et al. reported that people diagnosed with type D personality had a fourfold higher risk of developing sleep disorders and having type D was associated with sleeping fewer hours [53]. According to Domagalska et al., patients with a “stress” personality suffer from insomnia significantly more often [54]. Randler et al. studied the chronotypes in relation to the “Big Five” personality traits. According to the results of their research, extraversion, agreeableness, and conscientiousness have been associated with the morning chronotype, while neuroticism and openness to experience have been associated with the evening chronotype [55]. Thus, type D personality, which is equivalent to neurotic introversion, may be associated with changes in circadian activity [56]. As for neuroticism, it is known to be associated with less effective coping strategies [57].

High levels of stress can have serious consequences for students in terms of academic performance and dropout [58,59] and for mental health, including an increased risk of anxiety and depression [60]. For students, stress may be caused by socio-demographic, personal, and academic factors [61,62,63]. In particular, the chronotype—reflecting individual differences in circadian rhythms—can be an important factor in individual differences that govern the perception of stress. The authors of the study observed a significant negative correlation between the chronotype (arranged in order from evening to morning) and the scale of perceived stress and depression among the group of physical therapy students participating in the study. As indicated by the collected data, people diagnosed with evening and definitely evening chronotypes, scored higher in both scales, which proves their stronger perception of stress and a higher risk of depression. Similar conclusions have been made in the studies by Romo-Nava who observed that female academic stress and evening chronotype were the factors associated with an increased risk of developing a depressive episode [64]. Additionally, Supit et al. have demonstrated that people with an evening chronotype are more likely to have a high depression score [65]. Gorgol et al. came to similar conclusions, observing that high neuroticism, low conscientiousness, and low alpha stability are the factors that increase this risk, especially among the evening chronotypes [66]. The link found among the students may be due to the fact that classes usually start early in the morning, which may lead to a greater perception of stress among students with an evening chronotype, potentially because of greater negative affect and lower morning vigilance [67,68]. Other authors have also come to similar conclusions [58,69].

Psychological factors and specific stressors play a significant role in the development of TMDs [70,71]. The studies indicate that TMD patients report more life stressors than healthy people. People exposed to stress are believed to have a greater risk of the onset and progression of TMDs, and that stress and distress in life are more common in patients with TMDs [72]. The studies carried out to date have revealed that two important risk factors for temporomandibular joint disorders include sleep quality and stress level [73,74]. The authors of the studies came to similar conclusions, according to the collected data, a greater number of stressors in people with type D personality was linked with a greater number of reported TMDs symptoms, as compared with the control group (*p* < 0.001). The most frequently reported TMDs symptoms and predisposing factors in people with stressful personality disorder included: headache (80%), tooth clenching (67.2%), pain in the neck and shoulder girdle (61.88%), TMJ acoustic symptoms (45.4%), and increased masticatory muscle tension (38.1%). The most frequently reported stressors in students with type D personality included: loads of learning (63.6%), tests/exams (58.2%), “I think I can’t manage” (50%), and being tested verbally in front of the group (48.2%).

In the research carried out by the authors, the univariate logistic regression analysis showed that type D personality was associated with a significantly more frequent occurrence of all the analyzed TMDs symptoms, i.e., headache (OR 5.78), pain in the neck and shoulder girdle (OR 2.52), TMJ pain (OR 2.49), TMJ acoustic symptoms (OR 4.26), TMJ blocking (OR 4.15), increased masticatory muscle tension (OR 2.21), teeth clenching (OR 5.48), and teeth grinding (OR 2.67). Moreover, respondents diagnosed with type D personality more often experienced sleep deprivation, disappointment with studies, critical comments from the lecturer and difficulties in establishing contacts, a sense of not being understood, “unexpected situations”, and “speaking during classes”. Additionally, the authors of the study observed a significant influence of the definitely and moderately evening chronotype on the occurrence of type D personality. Among the potential confounding variables, a significant influence of the female gender and mild and moderate degree of depression on the occurrence of type D personality has been observed. Whereas, in the multivariate model, adjusted with the above-mentioned factors, an increased risk of type D personality has been observed in the case of the definite and moderate evening chronotypes, which turned out to be a significant factor for the occurrence of type D personality after adjusting with the remaining confounding variables.

Available data on the relationship of personality with chronotype, depression, and gender are still scarce. According to the research of Hidalgo et al., patients with the evening and late evening chronotypes have a greater chance of developing depressive symptoms compared to the morning and intermediate chronotypes. Additionally, it was found that an independent co-factor associated with a higher level of depressive symptoms was the female sex [75]. Similar conclusions were also reached by Kim et al., however, their study outcome concluded that late chronotype is associated with an increased risk of depression in women, but not in men. Yet, early chronotype was not significantly associated with depression in both sexes [76].

O’Riordan et al. showed that type D students perceived their life events as significantly more stressful than those without type D. They were also reported to have an increased perception of negative social relationships and less social support [77]. Similar conclusions were reached by Cho et al., after carrying out an analysis among Korean students. They observed that the type D personality is related to the level of experienced stress [78]. There are only a few studies in the literature regarding the analysis of the link between TMDs and the chronotype profile. In a study by Kirsrslan et al., it was observed that TMDs-related ailments (pain in the face, head, neck) occurred more often in students with an evening profile [79]. Additionally, an association was found between awake bruxism (AB) and the evening chronotype profile (*p* = 0.009). Similar conclusions were drawn by Serra-Negra et al. in studies conducted among dentistry students, the one-dimensional analysis showed that people with an evening chronotype profile were susceptible to AB [80]. The research carried out on Lithuanian students of dentistry also indicated that teeth grinding during the day related significantly to the evening chronotype (*p* = 0.039) [81]. Ribeiro et al. reported that children with the evening chronotype had a tendency to possible SB [82].

Scientific reports and the results obtained in this study confirm the proven predictive role of type D personality in the occurrence of a given type of circadian activity and its part in determining the level of stress perceived and developing TMDs. Therefore, it seems advisable to carry out psychological tests among students early on and to implement intervention programs among groups of young adults diagnosed with a “stress” personality type. The promotion of preventive measures in the aspect of preventing TMDs should be of key importance in people diagnosed with type D personality.

### Limitations

The presented results should be approached with some caution. It is mainly due to the cross-sectional nature of the research, which is why the results cannot be applied directly to the entire population. It would be also very difficult to establish a tangible causal relationship between all of our study parameters. It should be emphasized that the manifestation of a given chronotype may also depend on a few additional parameters, including: genetic predispositions, sex, age, socioeconomic factors, exposure to light, and the season of birth [1]. In addition, an individual’s personality is only one of the many factors related to TMDs development. An important limitation of the studies presented herein is the lack of physical examination of TMJs in order to objectively assess the presence of typical dysfunction. Moreover, it should be noted that a result obtained in the Beck Depression Inventory (BDI), used for self-assessment of well-being, is only an indication and not yet a diagnosis of depression. Yet, perceived stress was assessed solely on the basis of self-description, and future research should take into account its’ biological markers e.g., hormone levels.

Therefore, continuity of future research on the topic is warranted, with a physical examination of the masticatory organ and a thorough analysis of factors affecting the chronotype included. Additionally, the use of more precise psychological tools and extended diagnostics of stress assessment is considered mandatory by the writing team.

## 5. Conclusions

1. The evening chronotype, female sex, and symptoms of TMDs are associated with the type D personality. People presenting this chronotype have a higher level of perceived stress and depression.
2. Type D personality is associated with more frequent and numerous symptoms of TMDs and predisposing factors.
3. Students with an evening chronotype and type D personality may find it difficult to actively participate in morning activities.

## Figures and Tables

**Table 1:** Statistical analysis of the chronotypes within the G1 and G2 groups.

Chronotype Score	No Type D Personality (G2)		Type D Personality (G1)		*p*	OR	95% Cl
	*n*	%	*n*	%			
0	1	0.9	31	28.2	0.00	163.86	20.64
1	18	16.4	58	52.7	0.00	17.03	7.82
2	74	67.3	14	12.7	-	1.00	-
3	15	13.6	5	4.5	0.34	1.76	0.55
4	2	1.8	2	1.8	0.11	5.29	0.69

*p*—statistical significance; OR—odds ratio; 95% Cl—95% confidence interval. Legend of the chronotype score: 0—definitely evening; 1—moderately evening; 2—intermediate; 3—moderately morning; 4—definitely morning.

**Table 2:** Statistical analysis between the type of chronotype, the level of stress, and the occurrence of depression.

Variable	All Subjects		No Type D Personality (G2)		Type D Personality (G1)	
	Tau	*p*	Tau	*p*	Tau	*p*
PSS10 (sten)	−0.51	0.00	−0.27	0.00	−0.24	0.00
PSS10 (point)	−0.53	0.00	−0.28	0.00	−0.27	0.00
BDI	−0.63	0.00	−0.37	0.00	−0.49	0.00

*p*—statistical significance; Tau—Kendall′s tau, sten—unit of the psychological scale.

**Table 3:** Analysis of stressful situations and temporomandibular disorders (TMDs) symptoms and predisposing factors.

Stressful Situation	No Type D Personality (G2)		Type D Personality (G1)		All Subjects	
	*n*	%	*n*	%	*n*	%
1. relocation out of hometown	10	9.1	11	10.0	21	9.5
2. problem with commuting to the university	16	14.5	13	11.8	29	13.2
3. lare amount of learning	61	55.5	70	63.6	130	59.1
4. sleep deprivation	84	76.4	56	50.9	140	63.6
5. I do not think I can manage to do it	64	58.2	55	50.0	119	54.1
6. verbal examination in front of the group	44	40.0	53	48.2	96	43.6
7. tests/exams	65	59.1	64	58.2	129	58.6
8. waiting for the test/exam results	19	17.3	21	19.1	40	18.2
9. the perspective of repeating the year	21	19.1	17	15.5	38	17.3
10. fear of being expelled from the university	16	14.5	12	10.9	28	12.7
11. willingness to leave the studies	2	1.8	4	3.6	6	2.7
12. procrastination, postponing the studying process, and then panicking that there is too little time	33	30.0	38	34.5	70	31.8
13. disappointment with the studies	3	2.7	14	12.7	17	7.7
14. critical comments from the lecturer	9	8.2	27	24.5	36	16.4
15. difficulty in making contacts	4	3.6	29	26.4	33	15.0
16. conflicts with peers from the group	2	1.8	3	2.7	5	2.3
17. being laughed at/ironic comments from peers/friends	10	9.1	13	11.8	23	10.5
18. feeling of not being understood by anyone	15	13.6	27	24.5	42	19.1
19. unexpected situations	7	6.4	17	15.5	24	10.9
20. breaking up with a boyfriend/girlfriend	13	11.8	15	13.6	28	12.7
21. health problems	14	12.7	12	10.9	26	11.8
22. health problems in the family	22	20.0	33	30.0	55	25.0
23. speaking during classes	22	20.0	45	40.9	67	30.5
24. financial difficulties	23	20.9	22	20.0	45	20.5
25. conflicts with parents/guardians	5	4.5	8	7.3	13	5.9
26. lack of time for oneself	10	9.1	16	14.5	26	11.8
27. loneliness	14	12.7	25	22.7	39	17.7
28. being late for classes	5	4.5	4	3.6	9	4.1
29. relocation—change of residence in the hometown	3	2.7	3	2.7	6	2.7
TMJ symptoms and self-assessed predisposing factors						
Headache	45	40.9	88	80	133	60.5
Neck pain and/or shoulder girdle pain	43	39.1	68	61.8	111	50.5
TMJ pain	15	13.6	31	28.1	46	20.9
Acoustic symptoms in TMJ	18	16.4	50	45.4	68	30.9
TMJ locking	8	7.3	27	24.5	35	15.9
Increased masticatory muscles tension	24	21.8	42	38.1	66	30.0
Teeth clenching	30	27.3	74	67.2	104	47.3
Teeth grinding	20	18.2	41	37.2	61	27.7

*n*—population size; %—percent.

**Table 4:** Analysis of the number of stressors and the number of TMDs and predisposing factors in G1 and G2.

Variable	No Type D Personality (G2)			Type D Personality (G1)			*p*
	M_e_	Q1	Q3	M_e_	Q1	Q3	
Number of stressful situations	5.00	5.00	5.00	6.00	6.00	8.00	<0.001
Number of symptoms TMDs and predisposing factors	1.50	0.00	3.00	4.00	2.00	5.00	<0.001

M_e_—median; Q1—first quartile; Q3—third quartile; *p*—*p*-statistical significance.

**Table 5:** Univariate logistic regression analysis as regards the presence of the D personality.

Effect	Univariate Logistic Regression			
	*p*	OR	95% CI	
Number of stressful situations	0.00	1.84	1.53	2.20
Headache	0.00	5.78	3.16	10.55
Pain in the neck and shoulder girdle	0.00	2.52	1.47	4.34
TMJ pain	0.01	2.49	1.25	4.93
Acoustic symptoms in TMJ	0.00	4.26	2.27	7.99
TMJ locking	0.00	4.15	1.79	9.61
Increased masticatory muscles tension	0.01	2.21	1.22	4.01
Teeth clenching	0.00	5.48	3.07	9.78
Teeth grinding	0.00	2.67	1.44	4.97
Stressful situation				
1. relocation—moving out of hometown	0.82	1.11	0.45	2.73
2. problem with commuting to the university	0.55	0.79	0.36	1.73
3. large amount of learning	0.24	1.39	0.81	2.38
4. sleep deprivation	0.00	3.12	1.75	5.55
5. I do not think I can manage to do it	0.22	1.39	0.82	2.37
6. verbal examination in front of the group	0.25	1.37	0.80	2.34
7. tests/exams	0.89	0.96	0.56	1.65
8. waiting for the test/exam results	0.73	1.13	0.57	2.24
9. the perspective of repeating the year	0.48	0.77	0.38	1.56
10. fear of being expelled from the university	0.42	0.72	0.32	1.60
11. willingness to leave the studies	0.42	2.04	0.37	11.36
12. procrastination, postponing the studying process, and then panicking that there is too little time	0.53	1.20	0.68	2.12
13. disappointment with the studies	0.01	5.20	1.45	18.65
14. critical comments from the lecturer	0.00	3.65	1.63	8.19
15. difficulty in making contacts	0.00	9.49	3.21	28.07
16. conflicts with the peers from the group	0.65	1.51	0.25	9.24
17. being laughed at/ironic comments from peers/friends	0.51	1.34	0.56	3.20
18. feeling of not being understood by anyone	0.04	2.06	1.03	4.13
19. unexpected situations	0.04	2.69	1.07	6.77
20. breaking up with a boyfriend/girlfriend	0.69	1.18	0.53	2.61
21. health problems	0.68	0.84	0.37	1.91
22. health problems in the family	0.09	1.71	0.92	3.19
23. speaking during classes	0.00	2.77	1.52	5.06
24. financial difficulties	0.87	0.95	0.49	1.82
25. conflicts with parents/guardians	0.40	1.65	0.52	5.20
26. lack of time for oneself	0.21	1.70	0.74	3.94
27. loneliness	0.05	2.02	0.99	4.13
28. being late for classes	0.73	0.79	0.21	3.03
29. relocation change of residence in the hometown	1.00	1.00	0.20	5.07

*p*—statistical significance; OR—odds ratio; 95% Cl—95% confidence interval of the 95th percentile.

**Table 6:** Logistic regression analysis for type D personality occurrence.

Effect	Univariate Model				Multivariate Model			
	*p*	OR	95% CI		*p*	AOR	95% CI	
Age	0.74	0.98	0.86	1.11	0.65	0.95	0.75	1.20
Gender (female)	0.00	4.45	2.40	8.28	0.02	3.70	1.27	10.84
Mild depression	0.00	10.59	4.55	24.62	0.03	3.69	1.10	12.36
Medium depression	0.00	117.69	32.02	432.52	0.01	10.43	2.02	53.95
Chronotype 0 + 1	0.00	20.26	9.37	43.83	0.09	2.81	0.86	9.17
Chronotype 3 + 4	0.15	2.18	0.76	6.22	0.03	4.91	1.18	20.37
Sten PSS10	0.00	4.50	3.06	6.63	0.00	2.73	1.75	4.26

*p*—statistical significance; OR—odds ratio; AOR—adjusted odds ratio; 95% Cl—95% confidence interval of the 95th percentile; sten—the unit of the psychological scale. Legend of the chronotype: 0—definitely evening; 1—moderately evening; 3—moderately morning; 4—definitely morning.

## Data Availability

The data presented in this study are available upon request from the corresponding author. The data are not publicly available due to sensitive information.

## Supplementary Materials

The following supporting information can be downloaded at: https://www.mdpi.com/article/10.3390/jcm11071886/s1. Reference [83] is cited in the Supplementary Materials.
