# Supplementary material for: Chronotype Profile, Stress, Depression Level, and Temporomandibular Symptoms in Students with Type D Personality
Source: J Clin Med. 2022 Mar 28;11(7):1886. doi: 10.3390/jcm11071886 (PMC8999628; doi:10.3390/jcm11071886)
Supplement: Supplementary file 1 [file jcm-11-01886-s001.zip › jcm-1597881-supplementary.pdf]

Perceived Stress Scale (PSS10)

For each question choose from the following alternatives:
0 - never 1 - almost never 2 - sometimes 3 - fairly often 4 - very often

l. In the last month, how often have you been upset because of something that happened unexpectedly? _________

2. In the last month, how often have you felt that you were unable to control the important things in your life? _________

3. In the last month, how often have you felt nervous and stressed? _________

4. In the last month, how often have you felt confident about your ability to handle your personal problems? _________

5. In the last month, how often have you felt that things were going your way? _________

6. In the last month, how often have you found that you could not cope with all the things that you had to do? _________

7. In the last month, how often have you been able to control irritations in your life? _________

8. In the last month, how often have you felt that you were on top of things? _________

9. In the last month, how often have you been angered because of things that happened that were outside of your control? _________

10. In the last month, how often have you felt difficulties were piling up so high that you could not overcome them? _________

MORNINGNESS-EVENINGNESS QUESTIONNAIRE (MEQ)[1]

For each question, please select the answer that best describes you by circling the point value that best indicates how you have felt in recent weeks.

1. Approximately what time would you get up if you were entirely free to plan your day?
[5] 5:00 AM–6:30 AM *(05:00–06:30h)*
[4] 6:30 AM–7:45 AM *(06:30–07:45 h)*
[3] 7:45 AM–9:45 AM *(07:45–09:45 h)*
[2] 9:45 AM–11:00 AM *(09:45–11:00 h)*
[1] 11:00 AM–12 noon *(11:00–12:00 h)*

2. Approximately what time would you go to bed if you were entirely free to plan your evening?
[5] 8:00 PM–9:00 PM *(20:00–21:00h)*
[4] 9:00 PM–10:15 PM *(21:00–22:15 h)*
[3] 10:15 PM–12:30 AM *(22:15–00:30 h)*
[2] 12:30 AM–1:45 AM *(00:30–01:45 h)*
[1] 1:45 AM–3:00 AM *(01:45–03:00 h)*

3. If you usually have to get up at a specific time in the morning, how much do you depend on an alarm clock?
[4] Not at all
[3] Slightly
[2] Somewhat
[1] Very much

4. How easy do you find it to get up in the morning (when you are not awakened unexpectedly)?
[1] Very difficult
[2] Somewhat difficult
[3] Fairly easy
[4] Very easy

5. How alert do you feel during the first half hour after you wake up in the morning?
[1] Not at all alert
[2] Slightly alert
[3] Fairly alert
[4] Very alert

6. How hungry do you feel during the first half hour after you wake up?
[1] Not at all hungry
[2] Slightly hungry
[3] Fairly hungry
[4] Very hungry

7. During the first half hour after you wake up in the morning, how do you feel?
[1] Very tired
[2] Fairly tired
[3] Fairly refreshed
[4] Very refreshed

8. If you had no commitments the next day, what time would you go to bed compared to your usual bedtime?
[4] Seldom or never later
[3] Less that 1 hour later
[2] 1-2 hours later
[1] More than 2 hours later

9. You have decided to do physical exercise. A friend suggests that you do this for one hour twice a week, and the best time for him is between 7-8 AM *(07-08 h).* Bearing in mind nothing but your own internal "clock," how do you think you would perform?
[4] Would be in good form
[2] Would find it difficult
[3] Would be in reasonable form
[1] Would find it very difficult

10. At *approximately* what time in the evening do you feel tired, and, as a result, in need of sleep?
[5] 8:00 PM–9:00 PM *(20:00–21:00 h)*
[4] 9:00 PM–10:15 PM *(21:00–22:15 h)*
[3] 10:15 PM–12:45 AM *(22:15–00:45 h)*
[2] 12:45 AM–2:00 AM *(00:45–02:00 h)*
[1] 2:00 AM–3:00 AM *(02:00–03:00 h)*

11. You want to be at your peak performance for a test that you know is going to be mentally exhausting and will last two hours. You are entirely free to plan your day. Considering only your "internal clock," which one of the four testing times would you choose?
[6] 8 AM–10 AM *(08–10 h)*
[4] 11 AM–1 PM *(11–13 h)*
[2] 3 PM–5 PM *(15–17 h)*
[0] 7 PM–9 PM *(19–21 h)*

12. If you got into bed at 11 PM *(23 h),* how tired would you be?
[0] Not at all tired
[2] A little tired
[3] Fairly tired
[5] Very tired

13. For some reason you have gone to bed several hours later than usual, but there is no need to get up at any particular time the next morning. Which one of the following are you most likely to do?
[4] Will wake up at usual time, but will not fall back asleep
[3] Will wake up at usual time and will doze thereafter
[2] Will wake up at usual time, but will fall asleep again
[1] Will not wake up until later than usual

14. One night you have to remain awake between 4-6 AM *(04-06 h)* in order to carry out a night watch. You have no time commitments the next day. Which one of the alternatives would suit you best?
[1] Would not go to bed until the watch is over
[2] Would take a nap before and sleep after
[3] Would take a good sleep before and nap after
[4] Would sleep only before the watch

15. You have two hours of hard physical work. You are entirely free to plan your day. Considering only your internal "clock," which of the following times would you choose?
[4] 8 AM–10 AM *(08–10h)*
[3] 11 AM–1 PM *(11–13 h)*
[2] 3 PM–5 PM *(15–17 h)*
[1] 7 PM–9 PM *(19–21 h)*

16. You have decided to do physical exercise. A friend suggests that you do this for one hour twice a week. The best time for her is between 10-11 PM *(22-23 h).* Bearing in mind only your internal "clock," how well do you think you would perform?
[1] Would be in good form
[2] Would be in reasonable form
[3] Would find it difficult
[4] Would find it very difficult

17. Suppose you can choose your own work hours. Assume that you work a five-hour day (including breaks), your job is interesting, and you are paid based on your performance. At *approximately* what time would you choose to begin?
[5] 5 hours starting between 4–8 AM *(05–08 h)*
[4] 5 hours starting between 8–9 AM *(08–09 h)*
[3] 5 hours starting between 9 AM–2 PM *(09–14 h)*
*[2]*5 hours starting between 2–5 PM *(14–17 h)*
[1] 5 hours starting between 5 PM–4 AM *(17–04 h)*

18. At *approximately* what time of day do you usually feel your best?
[5] 5–8 AM *(05–08h)*
[4] 8–10 AM *(08–10 h)*
[3] 10 AM–5 PM *(10–17 h)*
[2] 5–10 PM *(17–22 h)*
[1] 10 PM–5 AM *(22–05 h)*

19. One hears about "morning types" and "evening types." Which one of these types do you consider yourself to be?
[6] Definitely a morning type
[4] Rather more a morning type than an evening type
[2] Rather more an evening type than a morning type
[1] Definitely an evening type

_____Total points for all 19 questions

[1]Horne JA and Östberg O. A self-assessment questionnaire to determine morningness-eveningness in human circadian rhythms. International Journal of Chronobiology, 1976: 4, 97-100.

Beck Depression Inventory (BDI)

1.
0 I do not feel sad.
1 I feel sad
2 I am sad all the time and I can't snap out of it. I am so sad and unhappy that I can't 3. stand it.
3 I am so sad and unhappy that I can't stand it.

2.
0 I am not particularly discouraged about the future.
1 I feel discouraged about the future.
2 I feel I have nothing to look forward to.
3 I feel the future is hopeless and that things cannot improve.

3.
0 I do not feel like a failure.
1 I feel I have failed more than the average person.
2 As I look back on my life, all I can see is a lot of failures.
3 I feel I am a complete failure as a person.

4.
0 I get as much satisfaction out of things as I used to.
1 I don't enjoy things the way I used to.
2 I don't get real satisfaction out of anything anymore.
3 I am dissatisfied or bored with everything.

5.
0 I don't feel particularly guilty
1 I feel guilty a good part of the time.
2 I feel quite guilty most of the time.
3 I feel guilty all of the time.

6.
0 I don't feel I am being punished.
1 I feel I may be punished.
2 I expect to be punished.
3 I feel I am being punished.

7.
0 I don't feel disappointed in myself.
1 I am disappointed in myself.
2 I am disgusted with myself.
3 I hate myself.

8.
0 I don't feel I am any worse than anybody else.
1 I am critical of myself for my weaknesses or mistakes.
2 I blame myself all the time for my faults.
3 I blame myself for everything bad that happens.

9.
0 I don't have any thoughts of killing myself.
1 I have thoughts of killing myself, but I would not carry them out.
2 I would like to kill myself.
3 I would kill myself if I had the chance.

10.
0 I don't cry any more than usual.
1 I cry more now than I used to.
2 I cry all the time now.
3 I used to be able to cry, but now I can't cry even though I want to.

11.
0 I am no more irritated by things than I ever was.
1 I am slightly more irritated now than usual.
2 I am quite annoyed or irritated a good deal of the time.
3 I feel irritated all the time.

12.
0 I have not lost interest in other people.
1 I am less interested in other people than I used to be.
2 I have lost most of my interest in other people.
3 I have lost all of my interest in other people.

13.
0 I make decisions about as well as I ever could.
1 I put off making decisions more than I used to.
2 I have greater difficulty in making decisions more than I used to.
3 I can't make decisions at all anymore.

14.
0 I don't feel that I look any worse than I used to.
1 I am worried that I am looking old or unattractive.
2 I feel there are permanent changes in my appearance that make me look unattractive
3 I believe that I look ugly

15.
0 I can work about as well as before.
1 It takes an extra effort to get started at doing something.
2 I have to push myself very hard to do anything.
3 I can't do any work at all.

16.
0 I can sleep as well as usual.
1 I don't sleep as well as I used to.
2 I wake up 1-2 hours earlier than usual and find it hard to get back to sleep.
3 I wake up several hours earlier than I used to and cannot get back to sleep.

17.
0 I don't get more tired than usual.
1 I get tired more easily than I used to.
2 I get tired from doing almost anything.
3 I am too tired to do anything.

18.
0 My appetite is no worse than usual.
1 My appetite is not as good as it used to be.
2 My appetite is much worse now.
3 I have no appetite at all anymore.

19.
0 I haven't lost much weight, if any, lately.
1 I have lost more than five pounds.
2 I have lost more than ten pounds.
3 I have lost more than fifteen pounds.

20.
0 I am no more worried about my health than usual.
1 I am worried about physical problems like aches, pains, upset stomach, or constipation.
2 I am very worried about physical problems and it's hard to think of much else.
3 I am so worried about my physical problems that I cannot think of anything else.

21.
0 I have not noticed any recent change in my interest in sex.
1 I am less interested in sex than I used to be.
2 I have almost no interest in sex.
3 I have lost interest in sex completely.

Total Score____________________

Our own questionnaire for assessing the occurrence of TMDs symptoms and self-assessed predisposing factors

Are you experiencing the following symptoms?. Mark the selected answer with an "X".

No.	Symptom	Yes	No
1	Headache		
2	Pain in the neck and shoulder girdle		
3	TMJ pain		
4	Acoustic symptoms in TMJ (e.g. clicking, crackling)		
5	TMJ locking (e.g. when opening and closing the mouth, chewing food)		
6	Increased masticatory muscles tension		
7	Teeth clenching		
8	Teeth grinding		

*TMJ – temporomandibular joint

Our own questionnaire for stressors during the course of studies

Select up stressors (maximum three). Mark the selected answer with an "X".

No.	Stressful situation	Yes
1	relocation	
2	problem with commuting to the university	
3	loads of learning	
4	sleep deprivation	
5	I do not think I can manage to do it	
6	examination in front of the group	
7	tests/ exams	
8	waiting for the test/ exam results	
9	the perspective of repeating the year	
10	fear of being expelled from the university	
11	willingness to leave the studies	
12	procrastination, postponing the studying process, and then panicking that there is too little time	
13	disappointment with the studies	
14	critical comments from the lecturer	
15	difficulty in making contacts	
16	conflicts with the peers from the group	
17	being laughed at/ ironic comments from peers/ friends	
18	feeling of not being understood by anyone	
19	unexpected situations	
20	breaking up with a boyfriend/ girlfriend	
21	health problems	
22	health problems in the family	
23	speaking during classes	
24	financial difficulties	
25	conflicts with parents/ guardians	
26	lack of time for oneself	
27	loneliness	
28	being late for classes	
29	relocation	
